# Sensing of Soil Organic Matter Using Laser-Induced Breakdown Spectroscopy Coupled with Optimized Self-Adaptive Calibration Strategy

**DOI:** 10.3390/s22041488

**Published:** 2022-02-15

**Authors:** Mengjin Hu, Fei Ma, Zhenwang Li, Xuebin Xu, Changwen Du

**Affiliations:** 1 The State Key Laboratory of Soil and Sustainable Agriculture, Institute of Soil Science, Chinese Academy of Sciences, Nanjing 210008, China; humengjin@issas.ac.cn (M.H.); fma@issas.ac.cn (F.M.); zwli@issas.ac.cn (Z.L.); xuxuebin@issas.ac.cn (X.X.); 2 College of Advanced Agricultural Sciences, University of Chinese Academy of Sciences, Beijing 100049, China

**Keywords:** soil organic matter, laser-induced breakdown spectroscopy, calibration strategy, self-adaptive model

## Abstract

Rapid quantification of soil organic matter (SOM) is a great challenge for the health assessment and fertility management of agricultural soil. Laser-induced breakdown spectroscopy (LIBS) with appropriate modeling algorithms is an alternative tool for this measurement. However, the current calibration strategy limits the prediction performance of the LIBS technique. In this study, 563 soil samples from Hetao Irrigation District in China were collected; the LIBS spectra of the soils were recorded in the wavenumber range of 288–950 nm with a resolution of 0.116 nm; a self-adaptive partial least squares regression model (SAM–PLSR) was employed to explore optimal model parameters for SOM prediction; and calibration parameters including sample selection for the calibration database, sample numbers and sample location sites were optimized. The results showed that the sample capacity around 60–80, rather than all of the samples in the soil library database, was selected for calibration from a spectral similarity re-ordered database regarding unknown samples; the model produced excellent predictions, with R^2^ = 0.92, RPD = 3.53 and RMSEP = 1.03 g kg^−1^. Both the soil variances of the target property and the spectra similarity of the soil background were the key factors for the calibration model, and the small sample set led to poor predictions due to the low variances of the target property, while negative effects were observed for the large sample set due to strong interferences from the soil background. Therefore, the specific unknown sample depended strategy, i.e., self-adaptive modelling, could be applied for fast SOM sensing using LIBS for soils in varied scales with improved robustness and accuracy.

## 1. Introduction

Soil functions as the largest carbon pool, and soil organic matter (SOM) plays a vital role in ecosystem, agricultural and land management [1,2,3,4,5]. Therefore, developing fast, efficient and accurate SOM content measurement methods is important for both agricultural production and environmental management. The traditional chemical method is time-consuming and expensive, making it difficult to meet the demands for the rapid and accurate monitoring of the extensive data in digital agriculture [6,7,8].

Spectroscopic methods have been introduced as rapid, in-situ and accurate alternative approaches for SOM sensing. Laser-induced breakdown spectroscopy (LIBS) is based on the atomic emission, and is a kind of modern spectroscopy technique for rapid soil property measurement, which has been used in quantitative analysis with acceptable measurement results [9,10,11,12,13,14,15,16]. Reliable chemometrics models are indispensable for spectroscopic methods, as they can maximize access to chemical property and spectral information by applying mathematics, statistics and computer science. The common chemometrics methods for soil analysis include multiple linear regression (MLR), partial least squares regression (PLSR), principal component analysis (PCA) and artificial neural networks (ANN), and they have achieved good predictions combining with LIBS [17,18,19,20].

Considering soil heterogeneity, several advanced and modified algorithms have been involved in the prediction of soil properties, which have produced good results. A self-adaptive PLSR model (SAM–PLSR) based on spectral similarity was proven to be an effective algorithm for SOM prediction in our previous work [21]. The spectral similarity between an unknown sample and each sample in a soil spectral database was calculated by the Euclidean distance (ED), and then the soil samples were sorted according to the ED-based similarity values, creating a new spectral data array; then, an optimal calibration dataset was built with appropriate parameters, such as RPD, R^2^ and RMSE, to predict soil properties, such as SOM. The above SAM–PLSR model, combined with mid-infrared photoacoustic spectra, was used for SOM prediction [21]. Compared with the conventional PLSR method, the values of R^2^ and RPD were increased by 1.60 and 1.96 times, respectively, and the RMSE value was reduced by almost 50%. The SAM–PLSR model combined with LIBS spectra was also used for predicting SOM, and the prediction accuracy was significantly improved compared with the conventional PLSR method [22]. Moreover, different spectral similarity calculation methods, such as correlation coefficients (CC), ED, Mahalanobis distance (MD), angle cosine (AC) and k-medoids (KM), have been used on various types of soil samples to determine the soil property prediction performance of the SAM–PLSR algorithm, and the SAM–PLSR based on ED and CC had the optimal model accuracy and robustness in the prediction of SOM; furthermore, a modified SAM–PLSR with LIBS spectra, based on individual distance and hybrid distance, was introduced [22], and the study also showed that the ED-based SAM–PLSR method showed good prediction performance. 

Besides the algorithm in modelling, the selection of the calibration dataset played a very important role in the prediction model, which depended on the database for calibration [23,24]. However, the predicted results varied due to different calibration databases [21,22]. Calibration datasets selected from one soil type at the county scale [21] and different types at the national scale [22] were investigated. When the variability of the soil samples was too small, the selected calibration dataset was not representative, which led to lower prediction accuracy; however, when the soil variability was too large, the increasing disturbance reduced the prediction accuracy. Therefore, it was found that the size of the calibration dataset should also be considered. As the number of samples in calibration datasets increased, the soil variability, calculation time and interference increased, and subsequently, the issue of how to build an optimal calibration dataset from a specific soil library database for unknown sample prediction remained unclear. 

Thus, the aim of this study was to explore the specific calibration strategy through selecting the optimal calibration dataset considering the balance between the variance of target soil property and the interferences from the soil background.

## 2. Materials and Methods

### 2.1. Study Area and Soil Sample Collection

**Figure 1:**
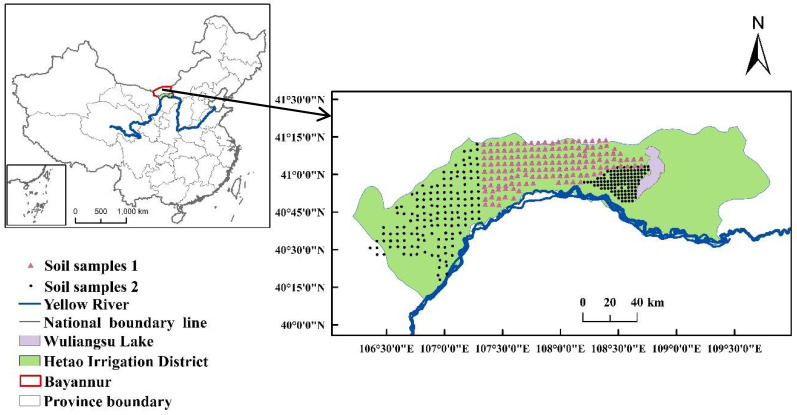
Study area in Hetao Irrigation District. Triangle denotes that 151 topsoil and the corresponding 151 subsoil samples were collected (soil samples 1), and circle denotes that 61 topsoil samples were collected (soil samples 2).

The study area was in Hetao Irrigation District, which is located in Inner Mongolia and along the Yellow River, China. Hetao Irrigation District has a typical mid-temperate continental climate. The soil types in this area are mainly loam, sandy loam and silt sand [25]. A total of 563 county-scale soil samples (412 from topsoil (0–20 cm) and 151 from subsoil (20–40 cm)) were collected, and a grid sampling design was set up for the collection with the resolution of 5 km × 5 km (Figure 1). The samples located in the center of the area were set up as Study Area 1, which contained 151 topsoil and 151 subsoil samples, and the samples surrounding these were deemed Study Area 2, composed of 261 topsoil samples. 

### 2.2. Soil Property Measurement

All soil samples collected from the study area were air-dried at an ambient temperature. Plant roots and other debris were removed, and then the samples were passed through a 2 mm sieve. Potassium dichromate oxidation colorimetry was used for the chemical determination of the SOM. Briefly, around 1.5 g of the soil sample was evenly mixed with 5.0 mL potassium dichromatic (K_2_Cr_2_O_7_, AR) solution (c (1/6 K_2_Cr_2_O_7_) = 0.8000 mol L^−1^) and 5.0 mL of concentrated sulfuric acid in a glass tube. The mixture was placed in an incubator (DRP-9162, China) at 100 °C for 90 min and was diluted with pure water to 50 mL after cooling. After 24 h, supernatants were used for colorimetry with standards at 590 nm using a spectrophotometer (BioTek Epoch, USA); then, the SOM content was obtained by multiplying the SOC by 1.724 [26]. The concentrations of Ca^2+^, Mg^2+^ and K^+^+Na^+^ were extracted with 1 mol L^−1^ ammonium acetate, and were determined by ICP–OES spectrometer (iCAP-7000, Thermo Fisher Scientific, Waltham, MA, USA), in which the atomic lines were 766.49 nm, 589.59 nm, 317.93 nm and 279.53 nm for K, Na, Ca and Mg, respectively [27]. The soil properties are listed in Table 1.

### 2.3. LIBS Spectra Measurement

The LIBS spectra of the soil samples were recorded with a MobiLIBS system (IVEA, France) with the AnaLIBS control software. The laser beam at 266 nm was obtained by the fourth-harmonic Nd: YAG laser (Quantel, France) with the pulse duration of 5 ns, the system frEquationuency was 20 Hz, and the delivery energy was 16 mJ. The spectral resolution was 0.116 nm, and the range of spectra was 288–950 nm. The delay time and the gate width were 137 μs and 7.0 ms, respectively. A 3 × 3 × 3 shot points at horizontal level and vertical level were set up for each sample pellet, and a total of 27 LIBS spectra were obtained from each sample. The LIBS spectra were preprocessed with baseline correction and normalization for solving the influence of spectral deviation and error caused by system instability, and the spectrum of each soil sample was obtained by the average spectrum of 27 spectra in each determination.

### 2.4. Self-Adaptive Model Partial Least Squares Regression (SAM–PLSR)

The process of the self-adaptive partial least squares model (SAM–PLSR) was applied to calculate the spectral ED between the unknown sample and the remaining samples in the soil library, and the soil samples database was further rearranged through ED from small to large. The ED was defined as Equation (1):

(1) $$ED_{ij}=\sqrt{\sum_{x=1}^{M}{\left(S_{xi}-S_{xj\;}\right)}^{2}}$$

where *ED_ij_* was the Euclidean distance of the *i*th unknown sample and the *j*th corresponding calibration sample; *i*, *j* = 1, 2, …, *M*, and *j* ≠ *i*. *x* was the spectral variable index; *x* = 1, 2, 3, …, *M*. *S* was the corresponding spectral intensity, which was standardized before calculation. Partial least squares regression (PLSR) was used to predict the soil properties. 

Considering that the range of maximal potential variable of the PLSR model was 20, the size of the calibration set should be more than 20. Thus, the initial sample set size was set as 20. The reordered soil samples were selected according to a certain interval value as calibration set for subsequent evaluation, and the interval was defined as 10 in the study, such as 20, 30, 40, 50, …, to the end of the database.

### 2.5. Model Evaluation Standard

The coefficients of determination (*R*^2^), the root mean square error (*RMSECP* and *RMSECV*), and the residual prediction deviation (*RPD*) and the ratio of *RMSEP/RMSEC* were used to evaluate the prediction models. The calculation formulas for the parameters were as follows:

(2) $$R^{2}=\frac{\sum_{i=1}^{N}{\left(y_{i}-\bar{y}\right)}^{2}}{\sum_{i=1}^{N}{\left(y_{i}^{’}-\bar{y}\right)}^{2}}$$

(3) $$RMSEP\left(RMSEC\right)=\sqrt{\frac{\sum_{i=1}^{N}{\left(y-y^{\prime }\right)}^{2}}{N}}$$

(4) $$RPD=\frac{SD}{RMSE}$$

where $y_{i}$ and $y_{i}^{’}$ were the ith predicted and measured values, respectively; $y_{\;i}^{’}$ was the mean value of measured values; and N was the number of samples. *SD* was the standard deviation of the measured values. Lower *RMSE* values, and higher *R*^2^ and *RPD* values indicated that the model was more robust and accurate. *RPD* was suitable for normally distributed data and the statistical distribution of the SOM of all soil horizons was normally distributed in this paper [28]. It has been classified that *RPD* < 1.4 indicates a poor model; *RPD* between 1.4 and 1.8 indicates fair model predictions; *RPD* values between 1.8 and 2.0 indicate a good model, where quantitative predictions are possible; *RPD* between 2.0 and 2.5 indicates very good quantitative model/predictions; and *RPD* > 2.5 indicates an excellent model [29]. *RMSEP/RMSEC* was another indicator for evaluating model quality, and for an excellent model, the value of *RMSEP/RMSEC* should be lower than 1.2 [29,30].

### 2.6. Software and Statistical Analysis 

The data were analyzed by MATLAB R2015b, and all figures were plotted by ArcMap 10.2.2 and Origin 2018.

## 3. Results

### 3.1. The Distribution of SOM 

**Figure 2:**
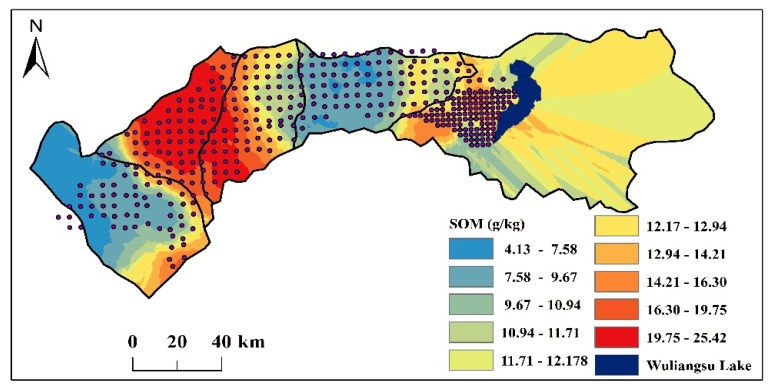
The distribution of the SOM from samples in Hetao Irrigation District (0–20 cm).

The distribution of the SOM and the statistics of the SOM from the samples are presented in Figure 2 and Table 1. The SOM content in the west part was low (<7.58 g kg^−1^); the region close to its east was the highest (>15.30 g kg^−1^); the place on the west of Wuliangsu Lake had SOM of 9.67–16.30 g kg^−1^. The SOM distribution was consistent with the soil types in Hetao Irrigation District, which were brown pedocals, cumulated irrigated soil, meadow solonchak soil and cumulated irrigated soil, respectively, from the west to the east. Moreover, in Table 1, the average value of the SOM in Study Area 1 was 9.11 ± 3.61 g kg^−1^ and 12.81 ± 6.08 g kg^−1^ in Study Area 2. The SOM content in the vertical samples was different as well, and the SOM values in topsoil (10.46 g kg^−1^) were higher than that in subsoil (7.74 g kg^−1^). Considering the variability, the CV value in Study Area 1 (39.63%) was smaller than that in Study Area 2 (47.46%), and the CV value of the sample of topsoil (32.98%) was smaller than that of subsoil (41.73%). 

### 3.2. The LIBS Spectra of Soil Samples

**Figure 3:**
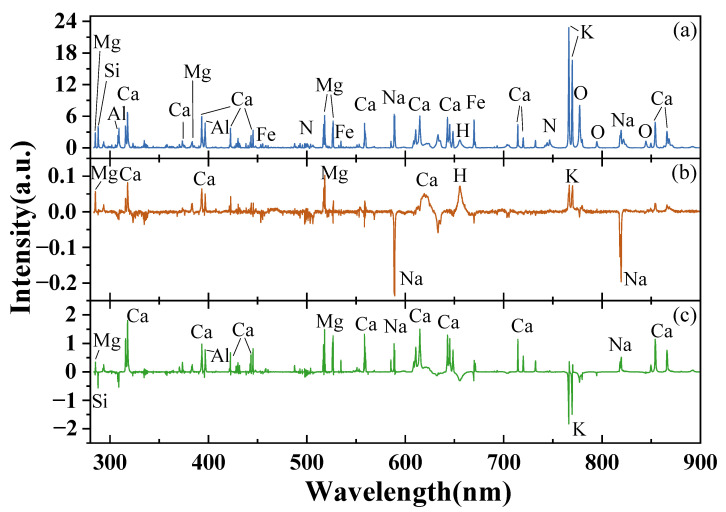
The LIBS spectra of soil samples. (**a**) The average spectra of all samples; (**b**) spectral difference of samples in topsoil and subsoil; (**c**) spectral difference of soil samples in the two study areas.

The average LIBS spectra from all soil samples are shown in Figure 3, and the main elements corresponding to Ca, Mg, Na, K, Fe, Al, Si, N, H and O in the soil samples were observed. Specifically, Figure 3b shows the spectral difference obtained by subtracting the subsoil from the topsoil, and it displays the peaks of Ca, Mg, Na, K and H, which means that the contents of Ca, Mg, Na and K were slightly different between the two sample depths. The average intensity of Mg, Ca, H and K in the topsoil was higher than that in the subsoil, while for Na, this was the opposite, which meant that Na-related compounds in the topsoil were lower than that in the subsoil. The differences between the samples in the two study areas are shown in Figure 3c. The variances, reflected by the peak and intensity, were mainly in the elements of Ca, Al, Mg, Na, Si and K. It was observed that the difference of elements between the two areas was almost 10 times that from the depths; moreover, Ca, Al, Mg and Na in Study Area 1 were higher than in area 2, but Si and K were lower. The mean values of Ca^2+^, Mg^2+^ and K^+^+Na^+^ are shown in Table 1, and it was found that the average content in the topsoil was higher than in subsoil. Meanwhile, higher content of Ca^2+^, Mg^2+^ and K^+^+Na^+^ were observed in Study Area 1, and the spectral qualifications were consistent with the results from the chemical analysis.

### 3.3. Optimization for Calibration Dataset

#### 3.3.1. Re-Ordered Spectra Database

SAM took soil spectral similarity as the selection criterion from the sample dataset, which meant that the model fully considered the spectral features of the soil. Firstly, the Euclidean distance of the spectra data between the unknown samples and the samples in the soil library database was calculated. Secondly, a new reordered database was constructed in terms of the value of spectral similarity from small to large for each unknown sample, and then a proper calibration dataset was built with the optimized sample sizes and model parameters.

#### 3.3.2. Optimal Sample Numbers for Calibration

For each unknown sample, the SAM–PLSR method was used to confirm the optimal sample number for calibration. Five parameters (SD, RMSEP, RPD, R^2^ and RMSEP/RMSEV) were used as the criteria for the optimal size of the calibration dataset.

**Figure 4:**
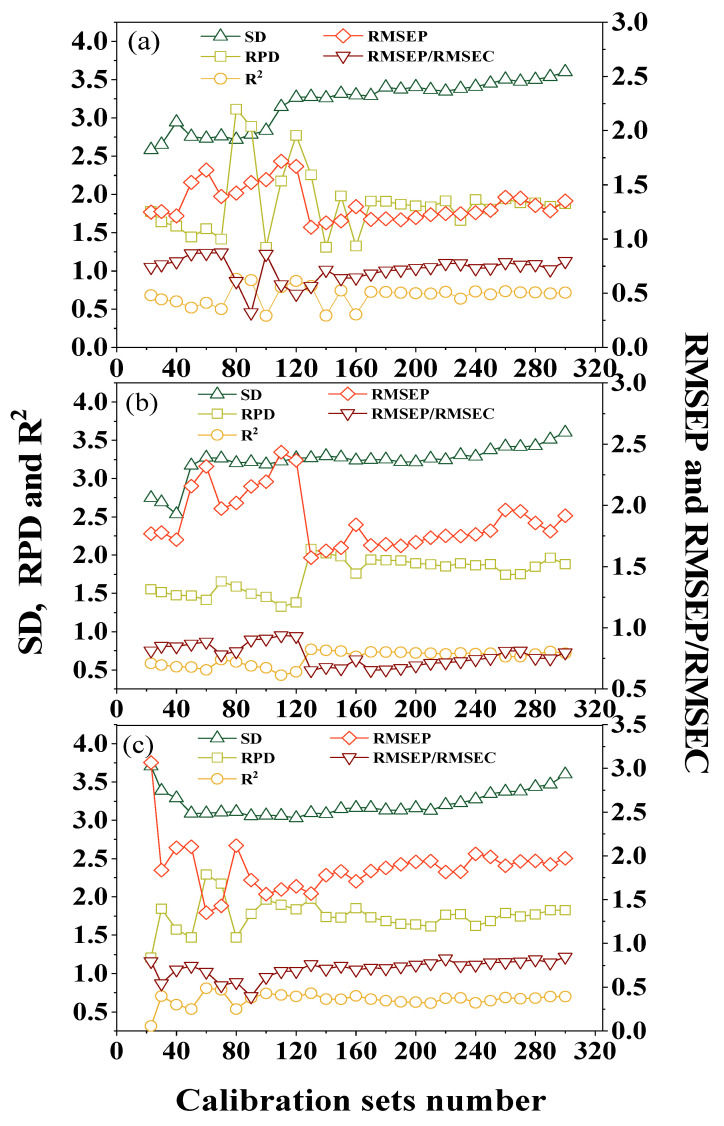
Evaluation of self-adaptive partial least squares regression prediction using the SD, RPD, R^2^, RMSEP and RMSEP/RMSEC of soil samples of various SOM content. (**a**) SOM content: 5.49 g kg^−1^; (**b**) SOM content: 9.61 g kg^−1^; (**c**) SOM content: 15.18 g kg^−1^.

Three soil samples representing low (5.49 g kg^−1^), medium (9.61 g kg^−1^) and high (15.18 g kg^−1^) SOM content were randomly selected as examples for explanation in detail. Three examples were deemed unknown samples to describe the building process of the calibration dataset. For the low SOM unknown sample, the SD, RPD, R^2^, RMSEP and RMSEP/RMSEC values of the SAM–PLSR model with various interval calibration numbers are showed in Figure 4a. The value of SD increased with the increase in the number of samples in the calibration dataset. The RPD, R^2^, RMSEP and RMSEP/RMSEC displayed a similar trend, with obvious fluctuation when the size of the calibration dataset was in the range of 70–160 samples, and remaining relative steady when the size of the calibration dataset was larger than 170 samples. The five parameters were optimized using the following criteria: the SD value should be larger, reflecting the heterogeneity of the selected samples; the RPD value should be greater than 2; the R^2^ value should be as large as possible and close to 1; RMSEP, which represents model accuracy, should be as small as possible; and the value of RMSEP/RMSEC should be less than 1.2 [29]. Thus, the optimal number for the calibration set for the sample was 80. For the medium SOM unknown sample shown in Figure 4b, similarly, the values of RPD, R^2^, RMSEP and RMSEP/RMSEC showed wide fluctuations when the size of the calibration dataset was between 40 and 170, while SD tended to remain stable and presented a trend of slow increase when the number of samples was larger than 50. The proper calibration dataset size for the sample was 130. For the high SOM unknown sample in Figure 4c, SD dropped rapidly when the sample numbers were less than 50, remained steady with samples between 50 and 130, and increased moderately with sample numbers over 130. RPD, R^2^, RMSEP and RMSEP/RMSEC fluctuated when the calibration numbers were less than 100; thus, the optimal sample number in this calibration dataset was around 60. 

**Figure 5:**
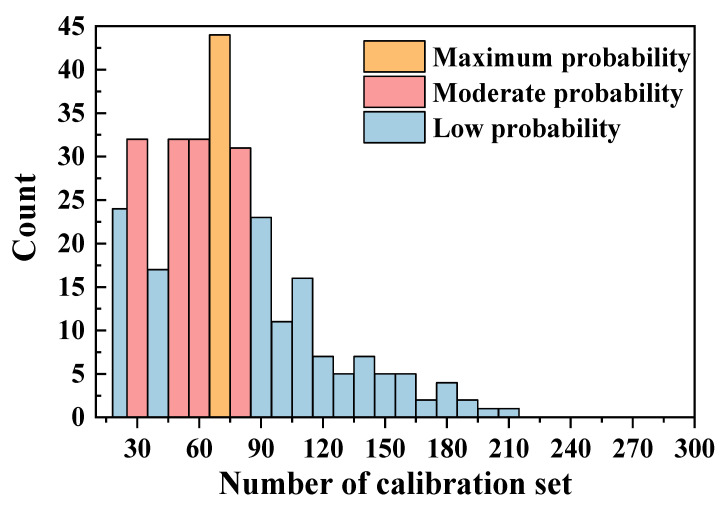
The statistics of the calibration set numbers for each unknown soil sample in the selection area using the SAM–PLSR method.

Figure 5 shows the statistics for the calibration set numbers. It was found that the maximum probability of the number of samples selected for the calibration dataset was about 70 (14.57%), followed by a sample size of 30 (10.63%), 50 (10.63%), 60 (10.63%) and 80 (10.26%), considering the optimal prediction results. Large numbers (more than 120) used for modeling have shown to have low probability (<2%), and larger numbers such as >210 were not selected for modeling due to the increased risk of interference in the predictions.

#### 3.3.3. Effects of Sample Depth on Calibration Dataset

**Figure 6:**
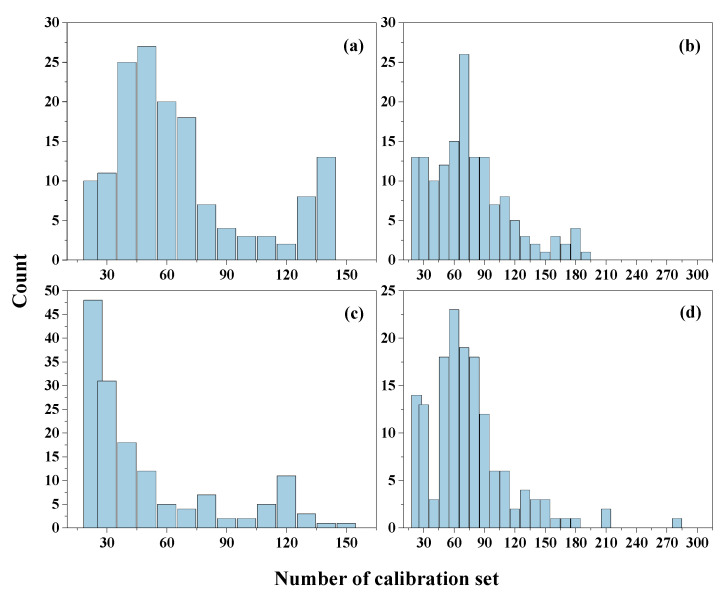
The statistics for the calibration set numbers of each unknown soil sample using the SAM–PLSR method: (**a**) unknown sample from top soil and calibration samples selected from top soil (0–20 cm) samples dataset; (**b**) unknown sample from top soil (0–20 cm) and calibration samples selected from 0–40 cm dataset; (**c**) unknown sample from subsoil (20–40 cm) and calibration samples selected from 20–40 cm dataset; (**d**) unknown sample from subsoil (20–40 cm) and calibration samples selected from 0–40 cm dataset.

As the spectra and SOM content of the soil samples from the topsoil and subsoil were different, the calibration datasets varied due to the spectral difference. The statistical analysis results of the selected samples from different depths are displayed in Figure 6. Samples from the two different depths were divided into two parts, and the unknown samples from the topsoil and the calibration dataset were only selected from the topsoil database and databases of both depths, respectively. Figure 6a,b show that the optimal numbers were 50 (proportion of 33.1%) and 70 (proportion of 23.2%), respectively. The unknown samples from the subsoil and the calibration dataset were only selected from the subsoil database and the databases of both depths, respectively, and the calibration samples in Figure 6c,d show that the respective optimal numbers were 23 (proportion of 15.2%) and 60 (proportion of 19.9%). 

#### 3.3.4. Effect of Sample Sites on Calibration Dataset

**Figure 7:**
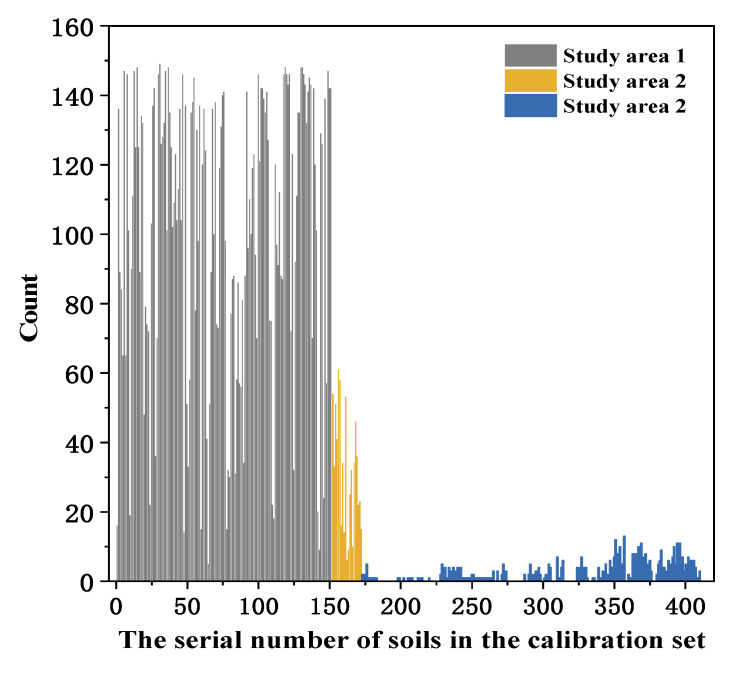
The frequency statistics of the selected samples according to SAM–PLSR. Study Area 2–1, the southeast section of Study Area 2; Study Area 2–2, the southwest section of Study Area 2.

The spectra of the samples in Study Areas 1 and 2 differed, and the effect of the sample site selection for the calibration dataset on the model results was noteworthy. The frequency statistics of the selected samples from the different study areas were plotted and are presented in Figure 7. Taking the unknown sample from Study Area 1 as an example, it was observed that the samples selected from this study area had the highest frequency, although all samples in the study area took part in the re-order modeling process. Moreover, samples with medium frequency were located in southeast section of Study Area 2 (Study Area 2–1), and samples in the southwest section of Study Area 2 (Study Area 2–2) had the lowest selection probability.

**Figure 8:**
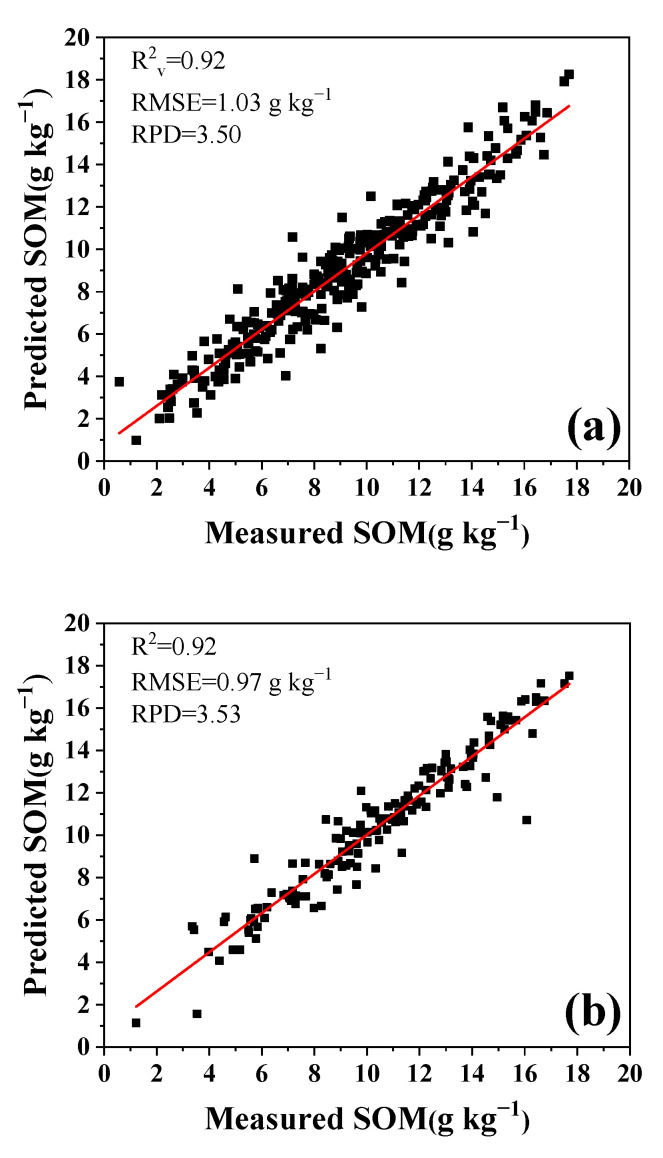

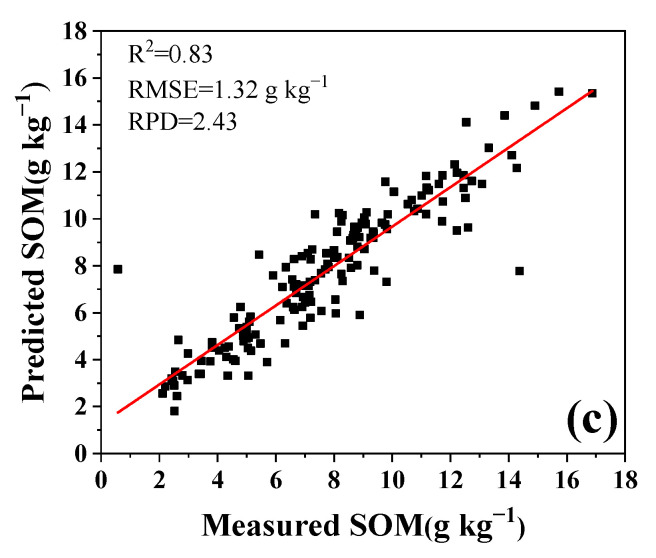
Scatterplot of predictions through the measured values and obtained by SAM–PLSR with calibration dataset of soils from different depths. (**a**) 0–40 cm depth; (**b**) 0–20 cm depth; (**c**) 20–40 cm depth.

Based on the above optimized parameters and calibration dataset, the prediction results with different calibration datasets were plotted and are shown in Figure 8. It was observed that using appropriate calibration samples selected from both the topsoil and subsoil datasets, SAM–PLSR demonstrated excellent performance with R^2^ = 0.92, RPD = 3.50 and lower RMSEP = 1.03 g kg^−1^ (Figure 8a). Using appropriate calibration samples only selected from the topsoil dataset, SAM–PLSR showed even better performance, with R^2^= 0.92, RPD = 3.53, and RMSEP =1.03 g kg^−1^ (Figure 8b). The calibration samples only selected from the subsoil dataset showed lower performance, with R^2^ = 0.83, RPD = 2.43, and RMSEP = 1.32 g kg^−1^ (Figure 8c). It was concluded that SAM–PLSR had the ability to reliably predict SOM. On the other hand, compared with the parameters from the calibration database with different soil depths, the calibration samples selected from the topsoil (0–20 cm) produced better evaluation than those from 0–40 cm and 20–40 cm. Although the values of R^2^ and RPD were similar, the RMSE value was lower than those from the 0–40 cm and 0–20 cm depth calibration databases.

Besides good prediction accuracy, the prediction efficiency and time cost should also be considered for an optimal model; as more samples are involved in the modeling, the prediction time and cost increase, and therefore preference should be given to samples that result in satisfactory predictions.

## 4. Discussion

### 4.1. LIBS Sensor for Soil Measurement 

The soil spectroscopy correlated with the soil composition and structure, which could be seen as the soil “fingerprint”. The LIBS spectra of the soil samples from different depths and areas differed, such as higher Ca, Mg and K content in topsoil than in subsoil due to soil salinization. The SOM varied in different areas because it was most affected by anthropogenic activities and soil types [31]. The results reflected by the spectra were consistent with the experimental results (Table 1), suggesting that LIBS is a fast and effective way to measure soil properties. It could be an alternative sensor for soil detection, especially for outdoor and in-situ environments where laboratory analysis is difficult. 

### 4.2. Influences on SAM–PLSR Modeling Performance

The algorithm was the first step for the spectra-based techniques, which decided the accuracy and robustness of the models. A SAM–PLSR method calculation has been proven effective considering the spectroscopy vector property. The spectral similarity arrangement could minimize the interference of irrelevant spectra and increase the effectiveness of the algorithm.

The prediction results suggested that the number of samples for calibration was important for an effective model, and not all of the samples in the soil library were suitable to be selected for calibration. A small number of samples had low variability, which led to low prediction accuracy, as the dataset was not representative. As the number of samples in the calibration dataset increased, the soil variability became larger; however, this introduced too much interference and reduced the accuracy of the results. Thus, soil variance was important for the soil calibration models, and large variances were good for building models [21]. Regarding the statistical results of the calibration dataset numbers in the study (Figure 5 and Figure 6), the appropriate number was about 60–80 from the re-ordered database, and it could be used for modeling and lead to excellent prediction results. The SD values in this range were large enough and did not increase too much. The sample selection from a topsoil depth of 0–20 cm for the calibration model produced the best results (Figure 6 and Figure 8). Although the values of CV of all samples at each depth were 32.98%, 41.73% and 39.63%, the SD values of the samples selected for calibration were 2.83, 2.41 and 2.96, which meant that 20 samples with high probability selected from the subsoil for calibration lacked soil variance, which led to the poorest predictions.

**Figure 9:**
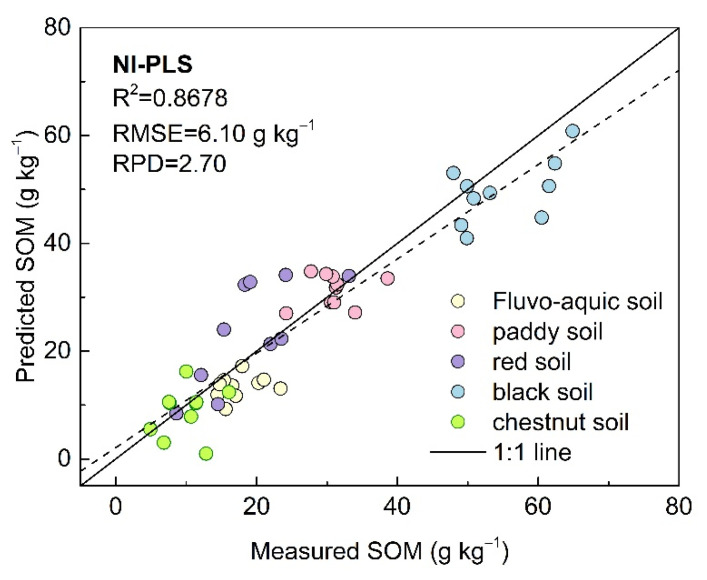
Scatterplot of prediction results and measured values obtained by SAM–PLSR at the national scale [22].

Sample collection from different study areas was also investigated. Usually, samples at the county scale should have spectral similarity. For example, the soil samples in Study Area 1 were mainly meadow solonchak soil, and the frequency statistics of the selected samples (Figure 7) suggested the samples in Study Area 1 were selected with higher probability for calibration than the unknown samples from the study, even if the SD and CV in Study Area 2 were larger than those in Study Area 1 (Table 1). In terms of larger sample selection for calibration at a national scale, 250 samples from all over the country were used with SAM–PLSR and LIBS for predicting SOM [22]; the results showed R^2^, RPD and RMSE values of 0.89, 2.82 and 5.84 g kg^−1^, respectively (Figure 9), which showed lower model performance compared with that in Figure 8. For a national database, the SD and CV of samples were 16.77 and 63.39%, respectively, which were larger than those of the samples in Study Area 1. 

Through comparative analysis of the three databases with different scales, including Study Area 1, Study Area 2 and the national scale, for the samples with the same soil type, differences in the variability of the SOM existed. However, when different types of soil were placed in the database across the country, the differences in the physical and chemical values of the soil were obviously too large. Although there were enough known samples in the national database to predict the SOM, the model effect obtained by SAM–PLSR was greatly reduced due to the huge number of calculations and high outlier interference; thus, spectral similarity should be highly considered as well as the variance of the target property. 

## 5. Conclusions

LIBS is a rapid method of soil analysis, and the calibration model was significant for the spectra-based sensor. Although a proper algorithm could enhance the accuracy and robustness of the prediction results, the parameters of the calibration model, such as sample selection, sample number, sample location and their effects on prediction accuracy, need to be explored. SAM–PLSR models with LIBS spectra were used to predict the SOM in Hetao Irrigation District, and a certain number of samples (60–80) selected for calibration produced good predictions with R^2^ = 0.92, RPD = 3.53 and RMSEP =1.03 g kg^−1^ from a spectra similarity re-ordered database. A small number of samples led to low prediction accuracy due to weak representation of the target property, and large variances were good for model building, but too many samples introduced interference, which could reduce the accuracy. For similar saline soil types, topsoil produced the best prediction results; for the soil at the county and national scales, soil similarity was also important for the soil calibration model. Thus, both soil variances of the target property and similarity of the soil background were important factors for the modeling, and 60–80 county-scale unknown samples could produce accurate and robust predictions.

## Figures and Tables

**Table 1:** Statistics of soil properties.

		SOM	Ca^2+^	Mg^2+^	K^+^+Na^+^
	n	(g kg^−1^)	(mg kg^−1^)	(mg kg^−1^)	(mg kg^−1^)
Study Area 1	302	9.11 ± 3.61	120.70 ± 70.67	64.48 ± 53.11	350.98 ± 194.25
0–20 topsoil	151	10.46 ± 3.45	126.70 ± 73.76	66.09 ± 55.57	360.81 ± 243.83
20–40 subsoil	151	7.74 ± 3.23	109.12 ± 46.54	59.56 ± 41.83	353.3 ± 189.45
Study Area 2 soil	261	12.81 ± 6.08	116.92 ± 66.98	62.46 ± 47.03	284.08 ± 193.72

## Data Availability

Data sharing not applicable.
